# The NeVa Net stent-retriever – initial report of 20 cases from two high volume centres

**DOI:** 10.1007/s00701-025-06586-5

**Published:** 2025-07-15

**Authors:** Pervinder Bhogal, Marco Mancuso-Marcello, Rory Fairhead, Nadia Shah, Keng Siang Lee, Christos Nikola, Katherine Parkin, Giovanna Klefti, Levansri Makalanda, Ken Wong, Joe Lansley, Michael Przyszlak, Oliver Spooner, Branimir Čulo, Vladimir Kalousek

**Affiliations:** 1 Department of Basic and Clinical Neurosciences, Maurice Wohl Clinical Neuroscience Institute, Institute of Psychiatry, Psychology and Neuroscience (IoPPN), King’s College London, London, UK; 2 Department of Neurosurgery, National Neuroscience Institute, Singapore, Singapore; 3 Department of Interventional Neuroradiology, The Royal London Hospital, Whitechapel Road, London, E1 1BB UK; 4 The London School of Medicine and Dentistry, Queen Mary University, Turner Street, London, UK; 5 Department of Stroke, The Royal London Hospital, Whitechapel Road, London, UK; 6 Department of Interventional Neuroradiology, UHC Sisters of Mercy, Zagreb, Croatia

**Keywords:** NeVa NET, Mechanical thrombectomy, Stroke, Large vessel occlusion, First pass effect, Microfilter

## Abstract

**Background:**

Various different technologies have been developed to optimise the mechanical thrombectomy procedure and first pass recanalisation. We report our initial experience with the NeVa NET – a novel stent-retriever with built in distal microfiltration net.

**Materials and methods:**

We performed a retrospective review of our prospectively maintained databases to identify all patients treated with the NeVa NET 4 × 30 mm device. We recorded the baseline demographics, NIHSS, ASPECT score and clot characteristics, first pass and final eTICI scores, complications and 90 day mRS.

**Results:**

We identified 20 patients (50% male), average age 67.9 ± 16.2 yrs, and median NIHSS 18 (range 6–29). 30% of patients received tPA. Average clot length was 21.6 ± 8.9 mm and median ASPECT score 9 (range 5–10). In 18 cases the NeVa NET could be tracked into position, however, in 2 cases it could not be tracked into position within the 0,021inch microcatheter.

First pass recanalization (eTICI ≥ 2c) was seen in 94% (n = 17) and the average number of NeVa NET passes was 1. A final eTICI score of ≥ 2c was achieved in 17 patients (94%). There were no emboli to new territories. No cases of vessel rupture or dissections occured due to the NeVa NET. 50% of patients achieved mRS 0–2. SAH was seen in 31.3% and SICH in 6.3% of the 16 patients for whom a 24 h CT head was available.

**Conclusion:**

The NeVa Net stent-retriever is uniquely designed to prevent distal embolisation and our initial experience demonstrates a very high FPE and mFPE. We believe this is the first publication on the use of this novel technology and larger studies are warranted.

## Introduction

Numerous advancements in the devices used to perform Mechanical Thrombectomy have been made since the pivotal stroke thrombectomy trials were published in 2015. These have largely revolved around improvements in catheter design with large and super-bore aspiration catheters such as the Millipede (Perfuze Medical) entering the clinical market and more navigable and larger inner lumen balloon guide catheters e.g. Walrus (Q’apel) and Emboguard (JnJ MedTech). The principle changes in stent-retriever design have revolved around smaller devices, for more distal occlusions, and segmented designs, as seen with the Embotrap (JnJ MedTech), which are thought to be more atraumatic. Devices such as the ERIC (Terumo Neuro), Embotrap and Neva (Vesalio) have also added a distal ‘clot catcher’ to the stent with the aim of preventing distal embolisation during the thrombectomy procedure and increasing the First Pass Effect (FPE).

The NeVa NET is an advancement on the concept of distal protection with the aim of preventing both large and small fragments from being lost distally during the procedure. The NeVa NET is the first device to incorporate technology specifically aimed at preventing microemboli and hence potentially improve both recanalization and reperfusion. Here we report our initial experience using the NeVa NET.

## Materials and methods

A retrospective analysis was conducted using prospectively maintained databases from two comprehensive stroke centers to identify all patients who underwent first-line treatment with the NeVa NET stent retriever between November 2023 and November 2024 and met predefined inclusion and exclusion criteria. The decision to utilize the NeVa NET device as initial therapy was based on operator discretion and device availability at the time of the procedure.

### Study population

The inclusion criteria included:

- Age ≥ 18
- National Institutes of Health Stroke Scale (NIHSS) score > 5
- ASPECT score ≥ 3
- LVO on CT angiography
- Life expectancy of > 6 months
- The NeVa NET device was the first stent-retriever device attempted for the treatment of the identified intracranial occlusion

Patients were excluded if another device or technique (aspiration) was used as the initial strategy for the MT procedure or if there was an incomplete dataset e.g. missing images from the procedure (aside from the 90 day mRS or 24 h post thrombectomy CT).

All patients underwent plain CT and CT Angiogram from the arch to the vertex prior to the MT procedure. Patients were considered eligible for MT if the ASPECT score was ≥ 3, and the life expectancy was greater than 6 months (in the case of known underlying malignancy), with no upper limit on age. All patients were given IV tPA if they met the criteria.

### Endovascular procedure

Patients were treated with local or general anaesthesia per on a cases-by-case basis as per operator discretion.

Choice of ancillary devices used in combination with the NeVa device varied between the different operators. A Walrus (Q’Apel) balloon guide catheter and a distal aspiration catheter were the most often preferred options used. Alternatively, a distal aspiration catheter, typically a 6 Fr Sofia (Microvention) or Cereglide 71 (JnJ MedTech), was introduced via a standard guide catheter such as a NeuronMax (Penumbra). Procedures were routinely performed via the right common femoral artery with ultrasound-guided puncture.

Successful recanalisation was defined as final eTICI ≥ 2b (67%) with first pass effect (FPE) and modified FPE defined as eTICI ≥ 2c and ≥ 2b (67%) after the first thrombectomy attempt, respectively.

### Post-procedure

Post procedural imaging is routinely performed at 24 ± 6 h unless there is a sudden deterioration in the conscious level prompting an earlier scan. In cases of intracranial or extracranial stenting a CTA was also performed.

The 90-day mRS is recorded via telephone interview or clinic review by a trained stroke physician.

The data recorded included the demographics (age, gender, underlying medical conditions, admission NIHSS etc.), use of IV tPA, radiological findings including the ASPECT score prior to MT, clot location, endovascular procedural information, and relevant timings. The eTICI score was recorded after the first pass of the device and also at the end of the procedure alongside any complications and the 90-day mRS where available.

#### Device design

**Fig. 1:**
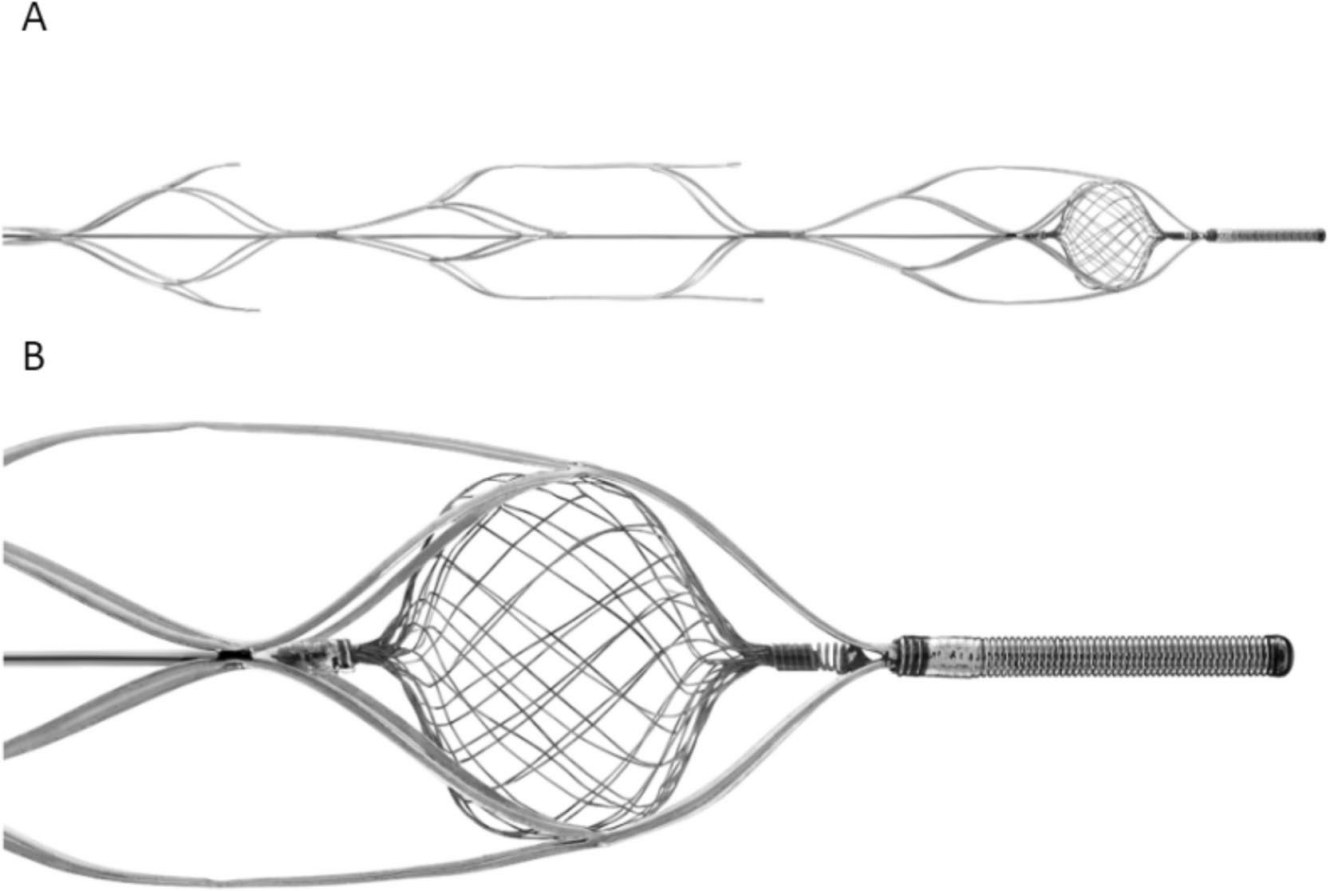
The NeVa NET device consisting of several offset ‘drop zones’ designed to allow clot integration into the stent **A**. The ‘NET’ component is designed to catch clot fragments, reducing distal microembolisation **B**

The construction of the NeVa NET is based on an outer laser-cut nitinol frame with a dual-layered nitinol-braid at the distal end of the device. The laser-cut frame has a proprietary pattern consisting of a series of pockets, referred to as ‘drop zones’, which are off-set to one another by 90 degrees as is seen in the standard NeVa device. The purpose of these drop zones is to allow clots, both hard and soft, to integrate into the stent (Fig. 1A). The additional dual-layered nitinol NET component was designed to prevent distal embolisation during the clot retrieval thereby optimising the mechanical thrombectomy procedure with the aim of increasing the first pass effect but also minimising distal microemboli (Fig. 1B). The NET is attached proximally to the pusher wire and distally it is attached to the inner surface of the nitinol frame. The NET is constructed from 32 individual strands of 0.00125 inch nitinol wire which have been braided to form a structure similar to a Web (Terumo Neuro). The clot facing pores of the NET, as with other similar structures, vary with the distance from the centre. Centrally the size of the openings is close to 0 and peripherally it is 400 microns. It is important to note that as the device is closed at both proximal and distal ends, the result of which is a double filter that captures micro-particles < 400 microns between the layers.

The device is currently available in two sizes – 5.5 × 37 mm and 4 × 30 mm. Both devices can pass through standard 0,027 inch microcatheters and the smaller can pass through 0,021 inch microcatheters.

The radial force of the 5.5 mm NeVa NET is similar to the Solitaire 6 × 30 mm device (Medtronic) across a range of relevant vessel diameters with the Solitaire having slightly higher radial resistive force and chronic outward force below 3.5 and 2.5 mm respectively.

## Results

### Baseline demographics, clinical results and angiographic results

The total number of MTs performed across the two participating sites in the study window was 663. We identified 20 patients that met our inclusion criteria, 50% of whom were male with average age 67.9 ± 16.2 yrs. The vast majority of patients were either mRS 0 or 1 (95%). The median NIHSS at presentation was 18 (range 6–29) and tPA was given prior to thrombectomy in 30% of patients. The most common cause of the LVO was thought to be cardioembolic. Left sided LVOs were more common in our cohort (60%) and there were two tandem lesions (10%). There was a single basilar occlusion. The majority of the occlusions were in the anterior circulation with proximal clot face in either the ICA (40%) or the M1 segment (50%). Hyperdense clots were seen in 70% of cases and the average clot length was 21.6 ± 8.9 mm. The median ASPECT score was 9 (range 5–10) (Table 1).

**Table 1:** Showing patient, stroke and clot characteristics

Baseline data	*n* = 20
**Demographics**	
Age	67.9
Female	10 (50%)
**Pre-morbid mRS**	
0	16 (80%)
1	3 (15%)
4	1 (5%)
**Stroke data**	
NIHSS	Median 18 (range 6–29)
IV tPA	6 (30%)
**Suspected cause**	
Cardioembolic	11 (55%)
Large Artery Atherosclerosis	2 (10%)
ICAD	3 (15%)
ESUS	3 (15%)
Dissection	1 (5%)
**Imaging findings**	
**Side**	
R	7 (35%)
L	12 (60%)
M	1 (5%)
**Tandem lesion**	
Y	2 (10%)
**Clot location**	
ICA	8 (40%)
M1	10 (50%)
VA	1 (5%)
BA	1 (5%)
**Hyperdense clot**	14 (70%)
**Clot length**	21.6 ± 8.9 mm
**ASPECT score**	Median 9 (range 5–10)

### Procedural outcomes

Local anaesthesia and conscious sedation were used in 90% of cases. In just over half of cases a BGC was used and distal aspiration catheter was used in all cases. In half of cases a 0,021 inch microcatheter was used to deploy the NeVa NET and in the other half of cases a larger 0,027 inch microcatheter was used. This reflected the differences in preference between the two sites.

In 18 cases the NeVa NET could be tracked into position however, in 2 cases it could not be tracked into position within the 0,021 inch microcatheter. This was felt to be due to tortuosity in the cervical ICA and cavernous ICA with a 360 loop in one and type 3 or 4 cavernous ICA in both cases. In these cases the standard NeVa 4.5 mm stentriever was used without issue in both cases. There were no reports of tracking difficulty when using the 0,027 inch microcatheter. The NeVa NET was typically deployed for approximately 4–5 min.

**Fig. 2:**
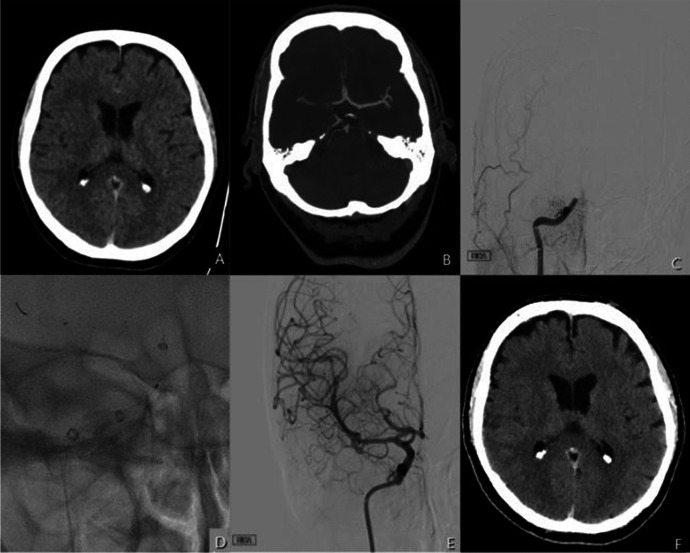
A patient in their 60 s presented with an NIHSS score of 12 and ASPECT score 6 **A**. After CTA confirmed an occlusion of the right terminal ICA and right proximal M1 **B**, the patient was transferred for MT. Initial angiography confirmed an abrupt filling defect of the right terminal ICA (**C**). A NeVa Net 4.0 × 30 mm was deployed for 5 min per our standard practice **D**. Angiography demonstrated eTICI 3 recanalization after the first pull **E**. The 24 h CT scan demonstrates expected hypodensity within the basal ganglia (ASPECT score 6) with no extension of the infarction when compared to the pre-procedural CT **F**

First pass recanalization (eTICI ≥ 2c) was seen in 17 cases (94%) and the average number of NeVa NET passes was 1. A final eTICI score of ≥ 2c was achieved in 17 patients (94%). Exemplar angiographic images are shown in Fig. 2. There were no emboli to new territories. Small very distal emboli, in distal M4 branches for example, within the same territory were seen in 5 cases (27.8%). There were no instances of changing to a different stentriever following an initial attempt with the NeVa NET. Carotid stenting was performed in 1 case (5%) and intracranial stenting for suspected ICAD was performed in 4 cases (20%).

There were no cases of vessel rupture or dissections due to either the procedure or the use of the NeVa NET (Table 2).

**Table 2:** Showing thrombectomy technique, equipment and immediate procedural outcomes

Procedural data	
**Anaesthesia**	
LA	18 (90%)
GA	2 (10%)
BGC	11 (55%)
Distal Aspiration Catheter	20 (100%)
**MicroCath**	
21	10 (50%)
27	10 (50%)
**Angiographic results**	
NeVa NET deployed	*n* = 18
Total no. of passes with Neva	1 (range 1)
	*n* = 18
FPE (eTICI 2c/3)	17 (94%)
Modified FPE (eTICI ≥ 2b)	17 (94%)
Final eTICI	
2a	1 (5.6%)
2b	0
2c	6 (33.3%)
3	11 (61.1%)
	*n* = 18
Distal embolisation	5 (27.8%)
Embolisation to new territory	0
	*n* = 20
Carotid Stent implanted	1 (5%)
Intracranial Stent implanted	4 (20%)

### Follow-up imaging and clinical results

CT imaging at 24 h was available for 16 patients with median ASPECT score of 7 (range 3–10). Subarachnoid haemorrhage without early neurological deterioration was seen in 5 of these patients (31.3%), all of whom had small, localised haemorrhage within the Sylvian fissure. Symptomatic intracranial haemorrhage, defined as an increase in NIHSS of ≥ 4 points, was seen in 1 patient (6.3%). The 90-day mRS was available for 16 patients with 8 patients (50%) achieving mRS 0–2, poor outcome of mRS 3–5 in 4 patients (25%), and mRS 6 in 4 (25%) (Table 3).

**Table 3:** Showing early and late outcomes

Follow-up (*n* = 16)	
**24 h post-MT ASPECT**	Median 7 (range 3–10) (*n* = 16)
sICH	1 (6.3%)
SAH	5 (31.3%)
**90 day post MT mRS**	(*n* = 16)
0–2	8 (50%)
3–5	4 (25%)
6	4 (25%)

## Discussion

This is the first publication looking at the clinical use of the 4 × 30 mm NeVa NET device, which shows it to have a high FPE and good safety profile. These initial recanalization results appear better than those seen with the standard NeVa device for which the average FPE from several studies is 51% [1, 3, 4, 8, 10]. The small sample size compared to the total number of MTs performed in the study period is predominantly driven by the availability of the device but is also perhaps a reflection of the understandably cautious process of novel device adoption into first line treatment strategies.

The NeVa NET is the only device available with a distal microfilter with the specific aim of capturing emboli and microemboli that may form during the MT procedure. Although the original NeVa device and the Embotrap have distal clot catchers, neither are able to trap smaller clots like the NeVa NET is able to. In the initial in vitro testing of the NeVa NET [2] it was shown, in comparison to a Solitaire (Medtronic), that the latter generated 4 times as many clot fragments > 1 mm compared to the NeVa NET (p = 0.037, Wilcoxan rank sum). With regards to smaller fragments, between 0.2–1 mm, the Solitaire produced 91 whereas the NeVa NET produced a total of 20 fragments and overall more fragments were generated during Solitaire thrombectomy than with NeVa NET thrombectomy (p = 0.048). In another study Li et al. [7] compared the NeVa NET (5.5 × 37 mm) with the Solitaire (6 × 40 mm) and Embotrap II (5 × 33 mm) in an in vitro model. A total of 150 MT attempts were made, 50 per device, and emboli measuring over 100 μm were collected and analysed after each case. In this study the rate of first pass recanalization across all devices was 52.7% with modified first pass recanalization achieved in 26.7% of attempts. This varied across devices (NeVa NET 66%, Solitaire 48% and Embotrap 44%) but did not meet statistical significance. The NeVa NET was successful at preventing distal embolisation with fewer emboli > 1 mm seen compared to the other devices (p = 0.003). Similarly, although there was no difference in the total number of emboli generated between the different devices these were smaller, in terms of total surface area, for the NeVa NET compared to the other devices (2.06 ± 1.85 mm^2^ vs. 4.06 ± 4.80 mm^2^, p = 0.013). A follow up study using the NeVa NET 4 × 30 mm device, and latest aspiration catheters and BGCs would be a welcome addition to the existing literature.

Although the exact effect distal small emboli have on clinical outcome is unquntified, it stands to reason that limiting microemboli during the procedure would be beneficial irrespective of their size. Indeed the recent CHOICE study [9] which evaluated the use of IA tPA following MT demonstrated a significant improvement in clinical outcome in the treatment arm, despite the fact that only ≈10% of patients showed an improvement in the eTICI grade pre- and post-IA tPA. One explanation for this may be the effect of the tPA on the microvascular perfusion and the results of CHOICE 2 are eagerly awaited. The NeVa NET may allow a lower dose of IA tPA to be used following the MT procedure as the surface area of emboli is approximately half of that produced with other devices. This would be an interesting study but may require an on-table CT perfusion scan at the end of the procedure which until recently was not feasible.

Although the NeVa NET 4 × 30 mm is designed to be compatible with 0,021 inch microcatheters we found that in two cases it could not be tracked into position. This was felt to have occurred secondary to tortuosity in the vessels, specifically the configuration of the cavernous ICA. We would advise that should pre-operative imaging show significant tortuosity in the ICA (loops and severe kinks) or of the cavernous ICA e.g. type 4 as described by Koge et al. [6] it may be advisable to use a 0,027 inch microcatheter as there have been no demosntrated issues with tracking these larger microcatheters. One downside to using a larger microcatheter is the slight increase in the number of microemboli generated during crossing of the clot [5]. However, as only a minority of the emboli are thought to occur as a result of this, it seems to be a reasonable trade-off for a high first pass recanalization rate and faster procedure in tortuous anatomy.

One potential indication where a single NeVa NET would still be ineffective in reducing distal embolisation or microembolisation is in the presence of a Y or T shaped clot. Although a double stent retriever technique could be used in these situations, we have not tried a double NeVa NET configuration in our experience to date. It of course remains conceivable that one arm of a Y or T shaped clot remains unretrieved during a pass with a single NeVa NET device resulting in distal embolisation.

The limitations of this study include that it is a retrospective study with a relatively small number of cases. An independent core lab did not score the eTICI scores and follow-up imaging and the use of other equipment varied depending on operator choice.

## Conclusion

The NeVa NET is a promising new device with a unique distal microfiltration system that enables a high first pass recanalization with no major safety concerns. Larger studies should be performed to corroborate these results.

## Data Availability

Research data supporting the findings in this paper is available upon request.

## References

[1] Akpinar CK, Ozdemir AO, Gurkas E et al (2021) Favorable first-pass recanalization rates with NeVa™ thrombectomy device in acute stroke patients: initial clinical experience. Interv Neuroradiol 27:107–113. 10.1177/1591019920938223 32615827 10.1177/1591019920938223 PMC7903549

[2] Anagnostakou V, Nogueira RG, Epshtein M et al (2022) Preclinical safety and efficacy of the NeVa NET™: a novel thrombectomy device with integrated embolic distal protection: preclinical safety and efficacy of the NeVa NET™. J Vasc Interv Neurol 14:1–16

[3] Bajrami A, Ertugrul O, Senadim S et al (2022) First pass results of mechanical thrombectomy with two-drop zone NeVaTM device. Interv Neuroradiol J Peritherapeutic Neuroradiol Surg Proced Relat Neurosci 15910199221135309. 10.1177/15910199221135309 10.1177/15910199221135309 PMC11475239 36314456

[4] Borggrefe J, Goertz L, Abdullayev N et al (2021) Mechanical Thrombectomy with the novel NeVa M1 stent retriever: do the drop zones represent a risk or a benefit? World Neurosurg 148:e121–e129. 10.1016/j.wneu.2020.12.075 33359523 10.1016/j.wneu.2020.12.075

[5] Caroff J, King RM, Arslanian R et al (2019) Microcatheter navigation through the clot: does size matter? J Neurointerventional Surg 11:271–274. 10.1136/neurintsurg-2018-014105 10.1136/neurintsurg-2018-014105 30177546

[6] Koge J, Tanaka K, Yoshimoto T et al (2022) Internal Carotid Artery Tortuosity: Impact on Mechanical Thrombectomy. Stroke 53:2458–2467. 10.1161/STROKEAHA.121.037904 35400203 10.1161/STROKEAHA.121.037904 PMC9311296

[7] Li J, Tiberi R, Bhogal P et al (2024) Impact of stent-retriever tip design on distal embolization during mechanical thrombectomy: a randomized in vitro evaluation. J Neurointerventional Surg 16:285–289. 10.1136/jnis-2023-020382 10.1136/jnis-2023-020382 37147003

[8] Masthoff M, Krähling H, Akkurt BH et al (2023) Evaluation of effectiveness and safety of the multizone NeVaTM stent retriever for mechanical thrombectomy in ischemic stroke. Neuroradiology 65:1777–1785. 10.1007/s00234-023-03236-4 37878032 10.1007/s00234-023-03236-4 PMC10654155

[9] Renú A, Millán M, San Román L et al (2022) Effect of intra-arterial Alteplase vs Placebo following successful Thrombectomy on Functional Outcomes in Patients With Large Vessel Occlusion Acute Ischemic Stroke: The CHOICE Randomized Clinical Trial. JAMA 327:826–835. 10.1001/jama.2022.1645 35143603 10.1001/jama.2022.1645 PMC8832304

[10] Ribo M, Requena M, Macho J et al (2020) Mechanical thrombectomy with a novel stent retriever with multifunctional zones: initial clinical experience with the NeVa™ thrombectomy device. J Neuroradiol J Neuroradiol 47:301–305. 10.1016/j.neurad.2019.03.007 30951765 10.1016/j.neurad.2019.03.007

